# Assessing the psychometric properties of generic (EQ-5D-5L) and disease-specific (KCCQ) quality of life in patients with hypertrophic cardiomyopathy in the AFFECT-HCM study

**DOI:** 10.1136/openhrt-2024-003143

**Published:** 2025-05-27

**Authors:** Isabell Wiethoff, Stephan A.C. Schoonvelde, Rudolf A. de Boer, Silvia M.A.A. Evers, Tjeerd Germans, Alexander Hirsch, Christian Knackstedt, Wouter P. te Rijdt, Marjon A. van Slegtenhorst, Arend F.L. Schinkel, Peter-Paul Zwetsloot, Michelle Michels, Mickael Hiligsmann

**Affiliations:** 1 Department of Health Services Research, Care and Public Health Research Institute (CAPHRI), Maastricht University, Maastricht, The Netherlands; 2 Department of Cardiology, Erasmus MC, Cardiovascular Institute, Thorax Center, Rotterdam, The Netherlands; 3 Trimbos Institute, Netherlands Institute of Mental Health and Addiction, Centre for Economic Evaluation, Utrecht, The Netherlands; 4 Department of Cardiology, Northwest Clinics, Alkmaar, The Netherlands; 5 Department of Radiology & Nuclear Medicine, Erasmus MC, Rotterdam, The Netherlands; 6 Department of Cardiology, Cardiovascular Research Institute Maastricht (CARIM), Maastricht University Medical Centre, Maastricht, The Netherlands; 7 Department of Clinical Genetics, Erasmus MC, Cardiovascular Institute, Rotterdam, The Netherlands; 8 Netherlands Heart Institute, Utrecht, The Netherlands

**Keywords:** Outcome Assessment, Health Care, Cardiomyopathy, Hypertrophic, Quality of Health Care

## Abstract

**Background:**

To assess the psychometric properties (content validity, reliability and construct validity) of generic and disease-specific health-related quality of life (HRQoL) instruments in patients with hypertrophic cardiomyopathy (HCM) and genotype-positive, phenotype-negative (G+/P−) individuals.

**Methods:**

As part of the multicentre, observational AFFECT-HCM study, HRQoL was measured using the generic EuroQoL-5 Dimension-5 Level (EQ-5D-5L) questionnaire, the Visual Analogue Scale (EQ VAS) and the disease-specific Kansas City Cardiomyopathy Questionnaire (KCCQ). The study included G+/P− individuals and HCM patients. EQ-5D-5L profiles were translated into EQ-5D values (utilities) using the Dutch value set. All instruments were evaluated regarding their general characteristics and health dimensions (content validity). Reliability was assessed using internal consistency (Cronbach’s alpha), response rate, floor/ceiling effects (percentage scoring highest/lowest), correlation and level of agreement between instruments (using Bland-Altman plots). Construct validity was assessed using the known-groups method to identify expected differences between relevant groups.

**Results:**

A total of 393 HCM patients and 78 G+/P− individuals were included in the psychometric assessment. Mean EQ-5D value in G+/P− individuals was 0.90 (81 EQ VAS, 93 KCCQ) and in HCM patients 0.84 (75 EQ VAS, 78 KCCQ). Ceiling effects were highest for EQ-5D values (51% in G+P; 32% in HCM), followed by the KCCQ (38% in G+P−; 12% in HCM) and the EQ VAS (8% in G+P−; 5% in HCM). KCCQ and EQ-5D values had the highest correlation (Spearman’s ρ=0.77) and showed good overall agreement according to the Bland-Altman plots. In HCM, EQ-5D values showed a slightly biased pattern with EQ-5D values scoring higher than the KCCQ. The KCCQ discriminated more nuances between relevant groups.

**Conclusions:**

Due to its simplicity and good overall agreement with the KCCQ—which showed slightly better discrimination—we propose from our data that the EQ-5D-5L is a suitable instrument for the HRQoL assessment in clinical practice in patients with HCM.

**WHAT IS ALREADY KNOWN ON THIS TOPIC**

- A regular assessment of the health-related quality of life (HRQoL) of genotype-positive, phenotype-negative individuals and hypertrophic cardiomyopathy (HCM) patients during routine care is important to improve the quality and patient-centredness of healthcare.
- An evaluation of the performance of the generic EuroQoL-5 Dimension-5 Level (EQ-5D-5L) descriptive system (including the EQ Visual Analogue Scale (VAS)) and the disease-specific Kansas City Cardiomyopathy Questionnaire (KCCQ) could provide insights into the eligibility of these instruments for clinical settings.

**WHAT THIS STUDY ADDS**

- The EQ-5D-5L, the EQ VAS and the KCCQ show an overall high level of correlation and agreement in HCM.
- All instruments discriminated between most known groups, with the KCCQ being most sensitive in distinguishing between more symptomatic clinical groups.

**HOW THIS STUDY MIGHT AFFECT RESEARCH, PRACTICE OR POLICY**

- Our study showed that the EQ-5D-5L can assess HRQoL with similar strength to the KCCQ.
- The EQ-5D-5L could, therefore, be a simple and short alternative for the routine HRQoL assessment in outpatient settings, whereas the KCCQ might be more prudent for clinical research.

## Introduction

 In hypertrophic cardiomyopathy (HCM), an inheritable cardiac disease characterised by left ventricular hypertrophy, patients often have reduced health-related quality of life (HRQoL) due to symptoms like chest pain, fatigue and syncope.^1^ Family members who do not have HCM (yet), but carry a (likely) pathogenic DNA variant, that is, genotype-positive, phenotype-negative (G+/P−) individuals, may also have impaired emotional well-being due to uncertainties and anxieties. A regular assessment of their HRQoL during routine care is important to improve the quality and patient-centredness of healthcare.^2 3^ The Kansas City Cardiomyopathy (KCCQ) questionnaire, a disease-specific instrument designed for the targeted assessment of the HRQoL of heart failure patients, has been widely used.^3^,^5^ However, it may have practical limitations for its application in clinical practice, as it is time-consuming and incurs costs for its usage.^6 7^ The generic EuroQoL-5 Dimension-5 Level (EQ-5D-5L) is being increasingly used due to its simplicity; however, it is also considered to be less nuanced in detecting smaller changes in HRQoL.^8^,^10^

The EQ-5D-5L consists of its descriptive questionnaire and the Visual Analogue Scale (EQ VAS).^9 11^ Due to its standardised character, EQ-5D values (also often referred to as utilities) can be derived, which represent societal-based health scores and allow a comparison between a wide range of diseases.^11^,^13^ Hence, the EQ-5D-5L is encouraged in various health economic guidelines for use in economic evaluations.^12 14^ Since generic instruments might be less sensitive in detecting changes in HRQoL, the EQ-5D-5L has not yet been frequently used in clinical practice. Previous literature on the performance of the EQ-5D-5L and the KCCQ in heart failure patients concluded that QoL findings remain consistent using both questionnaires.^15 16^ In a recent analysis of 9947 patients, the EQ-5D-5L showed a moderate-to-strong correlation with the KCCQ, and EQ-5D scores were robustly associated with hospitalisations and mortality.^17^ An evaluation of the performance of both instruments in HCM and G+/P− individuals could provide insights into whether the EQ-5D-5L is a suitable alternative for routine HRQoL monitoring in clinical settings.

The aim of this paper is to perform a psychometric assessment of the generic EQ-5D-5L descriptive system, the EQ VAS, and the disease-specific KCCQ. All instruments were analysed for general characteristics, content validity, reliability and construct validity in a cohort of G+/P− individuals and HCM patients.

## Methods

### Study design and patient population

This study used data from the multicentre, observational and prospective AFFECT-HCM study.^18^ Between November 2022 and December 2023, G+/P− individuals and HCM patients, aged 18–80 years, were recruited at three Dutch centres: Erasmus Medical Centre (Rotterdam), Maastricht University Medical Centre (Maastricht) and Northwest Hospital Group (Alkmaar). Participants were consecutively included at their corresponding hospital after assessing personal and family history and underwent physical examination, an ECG, and echocardiography if not otherwise performed during the last 6 months prior to inclusion. All participants were genotyped and divided based on phenotype into G+/P− individuals (maximal wall thickness <13 mm) and HCM patients. HCM patients were classified into non-obstructive HCM (nHCM, maximal provoked left ventricular outflow tract gradient <30 mm Hg) and obstructive HCM (oHCM, gradient ≥30 mm Hg). Furthermore, all HCM patients were stratified based on symptoms via the New York Heart Association (NYHA) functional classes.

### Patient and public involvement

Patient representatives contributed to the design of the AFFECT-HCM study by providing feedback during consortia meetings. Further, patients assisted in disseminating study information across their networks to promote participation.

### Quality of life measurement

Generic (EQ-5D-5L) and disease-specific HRQoL (KCCQ) were measured during study inclusion via self-completion using an electronic platform without investigator input. In the EQ-5D-5L, participants self-rated their health on the day of study inclusion. Overall health profiles were constructed for each participant, further translated into EQ-5D values (utility scores) that summarise the participants' HRQoL in a single numeric score.^11^ Therefore, country-specific value sets, that is, reference datasets of the general population, were used. In this study, the Dutch value set was applied.^13^ An EQ-5D value of one indicates perfect health while zero indicates a health state corresponding to death.^12^ EQ-5D values below zero represent health states worse than death.^11^ Additionally, participants self-rated their health status that day on the EQ VAS on a scale ranging from zero (worst health possible) to 100 (best health possible).^11^ Disease-specific HRQoL was measured with the 23-item version of the KCCQ.^5^ Based on the responses, an overall summary score (KCCQ-OS) was calculated, further transformed to values between 0 and 100, in which higher values reflect better health; lower scores represent more severe symptoms.^5^

### Psychometric properties

The EQ-5D-5L, the EQ VAS and the KCCQ were first assessed regarding their practicability and user-friendliness by contrasting their target group, number of items, outcome scores, response categories, reference period, mode of administration, completion time, languages and costs. Then, a psychometric evaluation was performed by selecting items of the COSMIN (Consensus-based Standards for the selection of Health Measurement Instruments) checklist.^19^

#### Content validity

According to COSMIN, content validity is the degree to which the content of an instrument adequately represents the full scope of the construct, here HRQoL, being measured.^19^ Therefore, the EQ-5D-5L and the KCCQ were compared regarding their items and health dimensions. Brazier *et al* published a list of physical and psychosocial health dimensions for the comparison of HRQoL instruments, which was adopted in this study to determine which of those dimensions are covered and how many items are devoted to each dimension.^20 21^

#### Reliability

The response rate was determined with the percentage of missing data for all instruments. Floor and ceiling effects were analysed by obtaining the frequency of patients scoring the highest and lowest possible scores. Internal consistency, that is, the degree of interrelatedness among items, was assessed with Cronbach’s alpha.^19^ To compare how well the instruments align in measuring HRQoL, correlation was assessed by means of the Spearman correlation coefficient. Further, the level of agreement was evaluated with Bland-Altman plots. To enable the comparison across instruments on the same scale, EQ-5D values were multiplied by 100. Potential bias between instruments was assessed with the mean difference between scores and by analysing the scatter pattern of the plots. Outliers were identified using the limits of agreements, defined as scatter points outside the 95% CI range of the mean difference. Plots were obtained for G+/P– individuals and HCM patients as well as for nHCM and oHCM patients to check whether the agreement differs between disease subtypes.

#### Construct validity

Due to the absence of a gold standard for HRQoL instruments, construct validity was examined by using the ‘known-groups’ method. This method applies hypothesis testing to assess the ability of an instrument to distinguish significant differences between prespecified groups and was used by several previous studies to investigate construct validity.^9 22^ Relevant groups were selected based on literature and discussed with the team. Lower HRQoL was expected in HCM patients (vs G+/P– individuals), symptomatic (vs asymptomatic) HCM patients and in higher NYHA classes, respectively. The difference in HRQoL between oHCM versus nHCM was tested without assuming a direction.

### Statistical analysis

Baseline characteristics are shown as frequencies (percentages) for categorical variables and mean±SD for continuous variables. Due to the high response rate, a complete case approach was chosen for the analysis. Mean differences between G+/P– individuals and HCM patients were analysed using the χ^2^ test for categorical data and the independent-samples t-test for continuous data. Minimum and maximum observed scores of all instruments were obtained and reported. Normality was analysed using histograms, QQ-plots, and the Shapiro-Wilk test. All mean scores were bootstrapped (2000 replications). Bootstrapped 95% CIs were derived with the percentile method. Subgroup analyses were performed in G+/P− individuals and HCM patients, nHCM and oHCM and according to NYHA class. To examine construct validity, one-sided Mann-Whitney U tests (two-sided for nHCM vs oHCM) were used. Mean differences between more than two groups were tested with the Kruskal-Wallis test and post hoc pairwise comparisons using Dunn’s test. P values were adjusted with the Bonferroni method. For all statistical tests, an alpha level of 0.05 was used. All analyses were performed in R V.4.2.2.

## Results

### Baseline data

The AFFECT-HCM study included 506 patients with a questionnaire response rate of 93%. Of the 506 participants, 84 (17%) were G+/P− individuals, with a mean age of 46 years (±14.8) and 68% being female, and 422 (83%) were HCM patients with a mean age of 57 (±13.6) and 33% being female. In total, 313 (74%) of the HCM patients were classified as nHCM and 109 (26%) as oHCM. Further, 254 (60%) patients, excluding G+/P− individuals, were in NYHA class I (asymptomatic) and 141 (33%) and 27 (6%) were in the symptomatic NYHA classes II and III, respectively. All baseline characteristics are summarised in online supplemental table S1.

### Practicability and user-friendliness

The EQ-5D-5L, the EQ VAS and the KCCQ differ vastly in their general characteristics, mainly due to their different purposes. A holistic assessment of the general characteristics, such as a comparison of their purpose, practicability and user-friendliness, is provided in table 1.

**Table 1:** General characteristics of the EQ-5D-5L, EQ VAS and KCCQ

Characteristics	EQ-5D-5L^11 25 28^	EQ VAS^11 28^	KCCQ^5 6 29 30^
Purpose			
Type	Generic	Generic	Disease-specific
Validated conditions	Various	Various	Heart failure; obstructive hypertrophic cardiomyopathy; valvular heart disease
Data collection purpose	General assessment of the health-related quality of life of patients, comparable between diseases	Holistic assessment of the health-related quality of life of patients, comparable between diseases	Targeted assessment of the impact of the conditions on the patient and identification of treatment needs
Reference data	Standardised descriptive system with societal based reference datasets (utilities)	Self-rated health score	Condition-specific comparisons to improve patient-centred care
Practicability (clinical practice/research)			
Target group (years)	Individuals ≥16 (youth version available)	Individuals ≥18	Individuals ≥18
Dimensions	5	1	7
Outcome scores	1 health profile/1 utility score	1 score	7 scores plus 2 summary scores
Available languages	>150	>150	>100
Costs	Free, registration required	Free, registration required	Paid, fee per patient
User-friendliness (patients)			
Number of items	5 (3-level version available)	1	23 (12-level version available)
Response categories	Categorical: 5-item Likert scale (no–extreme problems)	Visual: thermometer-like scale	Categorical: 1–5 to 1–7 item Likert scale (varying per question including opt out option)
Recall period	Today	Today	2 weeks
Mode of administration	Self-completion or interview (online and paper versions)	Self-completion or interview (online and paper versions)	Self-completion or interview (online and paper versions)
Completion time	<2 min for both		~ 4–6 min

EQ-5D-5L, EuroQoL-5 Dimension-5 Level; KCCQ, Kansas City Cardiomyopathy questionnaire; VAS, Visual Analogue Scale.

### Content validity

The KCCQ dedicates 13 of its 23 items to physical health. The EQ-5D-5L allocates four of its five items to physical health, with one item (usual activities) addressing both physical and psychosocial health (table 2). Both questionnaires cover the dimensions of mobility/physical activity, bodily function/self-care, pain/discomfort and usual activities (related to physical health). Furthermore, the KCCQ includes two items on vitality. Most items of the KCCQ are devoted to mobility/physical activity (five in total), including one item on symptom development over time. Regarding the list of dimensions for psychosocial health, the KCCQ covers more themes than the EQ-5D-5L. While the EQ-5D-5L covers the dimensions of well-being/depression/anxiety and usual activities, the KCCQ additionally includes items on sleeping, autonomy/control/dignity, relationships and social functioning/belonging and intimacy.

**Table 2:** Health dimensions of the EQ-5D-5L and the KCCQ

List of health dimensions (based on Brazier *et al*^21^)	KCCQ	Items	EQ-5D-5L	Items
Physical health				
Mobility/physical activity	✓	5	✓	1
Bodily function/self-care	✓	2	✓	1
Dexterity	–	–	–	–
Coping	–	–	–	–
Pain/discomfort	✓	2	✓	1
Senses (vision/hearing)	–	–	–	–
Usual activities/work/role related to physical health	✓	2	✓	1*
Vitality	✓	2	–	–
Psychosocial health				
Sleeping	✓	1	–	–
Well-being/depression/anxiety/happiness/calmness	✓	3	✓	1
Hope	–	–	–	–
Autonomy/control/dignity	✓	2	–	–
Self-esteem/security	–	–	–	–
Cognition/memory	–	–	–	–
Usual activities/work/role related to psychosocial health	✓	2	✓	1*
Relationships/social/functioning/belonging	✓	1†	–	–
Family	✓	1†	–	–
Intimacy (including sexual relations)	✓	1	–	–
Sum of items		23		5

* Dimension ‘usual activities’ (eg, work, household, family, leisure activities) considers physical as well as psychological reasons and is, therefore, put into both categories.

† Table 1 is based on the adapted list of health dimensions presented in Brazier *et al*^21^, originally published in Richardson *et al.*^20^ Item 22 of the KCCQ refers to the impact of the condition on meeting family and friends, hence the dimensions of family and relationships/social/functioning/belonging is relevant.

EQ-5D-5L, EuroQoL-5 Dimension-5 Level; KCCQ, Kansas City Cardiomyopathy Questionnaire.

### Reliability

#### Response rate and internal consistency

The response rate was high with a minimum of 5.0% and a maximum of 11.1% of missing observations in the different disease subgroups. Throughout all instruments, HRQoL was higher in G+/P− individuals compared with HCM patients (table 3). In G+/P− individuals, the KCCQ-OS and the EQ-5D value were highest with 92.6 (±11.5) and 0.902 (±0.1); the EQ VAS score was lowest with 80.6 (±12.4). In HCM and its subtypes nHCM and oHCM, the EQ-5D values were consistently highest across all instruments (HCM: 0.836±0.2; nHCM 0.839±0.2; oHCM: 0.828±0.2). With more severe NYHA classes, all HRQoL scores declined, respectively (p<0.001). In NYHA I (asymptomatic patients), EQ-5D values and the KCCQ-OS were highest (0.892±0.1 and 87.5±14.5), while the EQ VAS score was lowest (79.1±13.7). In NYHA II and III (symptomatic patients), the KCCQ-OS was on average lowest (65.3±20.7 and 50.4±21.6, respectively). Bootstrapped means did not differ from the observed sample data (online supplemental table S2). Both instruments demonstrated good internal validity with a Cronbach’s alpha of 0.797 (0.712; 0.797) for the EQ-5D-5L and 0.946 (0.938; 0.952) for the KCCQ.

**Table 3:** Baseline health-related quality of life data and response rate of AFFECT-HCM study

	n	EQ-5D value	EQ VAS score	KCCQ-OS
**Response rate—missing observations n (in %)**				
G+/P− individuals	84	6 (7.1%)	6 (7.1%)	6 (7.1%)
All HCM patients	422	28 (6.6%)	28 (6.6%)	29 (6.9%)
HCM subtype				
Non-obstructive HCM	313	21 (6.7%)	21 (6.7%)	21 (6.7%)
Obstructive HCM	109	7 (6.4%)	7 (6.4%)	8 (7.3%)
Symptomatology				
HCM and NYHA I	254	18 (7.1%)	18 (7.1%)	19 (7.5%)
HCM and NYHA II	141	7 (5.0%)	7 (5.0%)	7 (5.0%)
HCM and NYHA III	27	3 (11.1%)	3 (11.1%)	3 (11.1%)
**Health-related quality of life—mean scores (±SD)**				
G+/P− individuals	78	0.902 (0.1)	80.6 (12.4)	92.6 (11.5)
All HCM patients	393	0.836 (0.2)	74.9 (15.9)	77.7 (21.3)
HCM subtype				
Non-obstructive HCM	292	0.839 (0.2)	75.5 (15.8)	78.7 (21.1)
Obstructive HCM	101	0.828 (0.2)	73.2 (15.9)	74.8 (21.7)
Symptomatology				
HCM and NYHA I	235	0.892 (0.1)	79.1 (13.7)	87.5 (14.5)
HCM and NYHA II	134	0.769 (0.2)	69.7 (16.8)	65.3 (20.7)
HCM and NYHA III	24	0.662 (0.2)	62.5 (15.8)	50.4 (21.6)
Internal consistency		EQ-5D-5L	EQ-VAS	KCCQ
Cronbach’s alpha (95% CI)		0.761 (0.712; 0.797)	–	0.946 (0.938; 0.952)

EQ-5D, EuroQoL-5 Dimension; G+/P−, genotype positive, phenotype negative; HCM, hypertrophic cardiomyopathy; KCCQ-OS, Kansas City Cardiomyopathy Questionnaire–Overall Summary Score; NYHA, New York Heart Association class; VAS, Visual Analogue Scale.

#### Floor and ceiling effects

Across all instruments, no floor effects were reported (table 4). Two patients had an EQ-5D value below zero (−0.012 and −0.121), which is higher than the lowest possible score of −0.446 according to the Dutch value set. Ceiling effects were reached in all instruments across all subgroups, except from NYHA III, with most ceiling effects in EQ-5D values. In G+/P− individuals, 50% of the participants reached a perfect score; 31.5% in HCM patients. Lowest ceiling effects were found in the EQ VAS scores with 7.7% in G+/P− individuals and 4.8% in HCM patients. In the KCCQ-OS, ceiling effects were highest in G+/P− individuals with 37.2%. For HCM patients, ceiling effects dropped to 11.9%. In NYHA II, the KCCQ-OS had the lowest ceiling effects with 0.8%.

**Table 4:** Floor/ceiling effects of the EQ-5D values, EQ VAS scores and the KCCQ-OS

Lowest and highest reported scores	n	EQ-5D value		EQ VAS score		KCCQ-OS	
		Min	Max	Min	Max	Min	Max
G+/P− individuals	78	0.340	1.0	36	100	50.0	100
All HCM patients	393	−0.121	1.0	11	100	10.1	100
HCM subtype							
Non-obstructive HCM	292	−0.012	1.0	11	100	10.1	100
Obstructive HCM	101	−0.121	1.0	27	100	11.2	100
Symptomatology							
HCM and NYHA I	235	0.387	1.0	11	100	39.6	100
HCM and NYHA II	134	−0.012	1.0	21	100	10.2	100
HCM and NYHA III	24	−0.121	0.918	27	89	10.2	81.3
Floor and ceiling effects in %	n	Floor	Ceiling	Floor	Ceiling	Floor	Ceiling
G+/P− individuals	78	0%	50.0%	0%	7.7%	0%	37.2%
All HCM patients	393	0%	31.5%	0%	4.8%	0%	11.9%
HCM subtype							
Non-obstructive HCM	292	0%	32.5%	0%	5.1%	0%	12.7%
Obstructive HCM	101	0%	28.43	0%	3.9%	0%	9.9%
Symptomatology							
HCM and NYHA I	235	0%	45.2%	0%	7.0%	0%	24.0%
HCM and NYHA II	134	0%	15.7%	0%	2.2%	0%	0.8%
HCM and NYHA III	24	0%	0%	0%	0%	0%	0%

EQ-5D value, EuroQoL-5 Dimension; G+/P−, genotype positive, phenotype negative; HCM, hypertrophic cardiomyopathy; KCCQ-OS, Kansas City Cardiomyopathy Questionnaire–Overall Summary Score; NYHA, New York Heart Association class; VAS, Visual Analogue Scale.

#### Correlation

**Figure 1:**
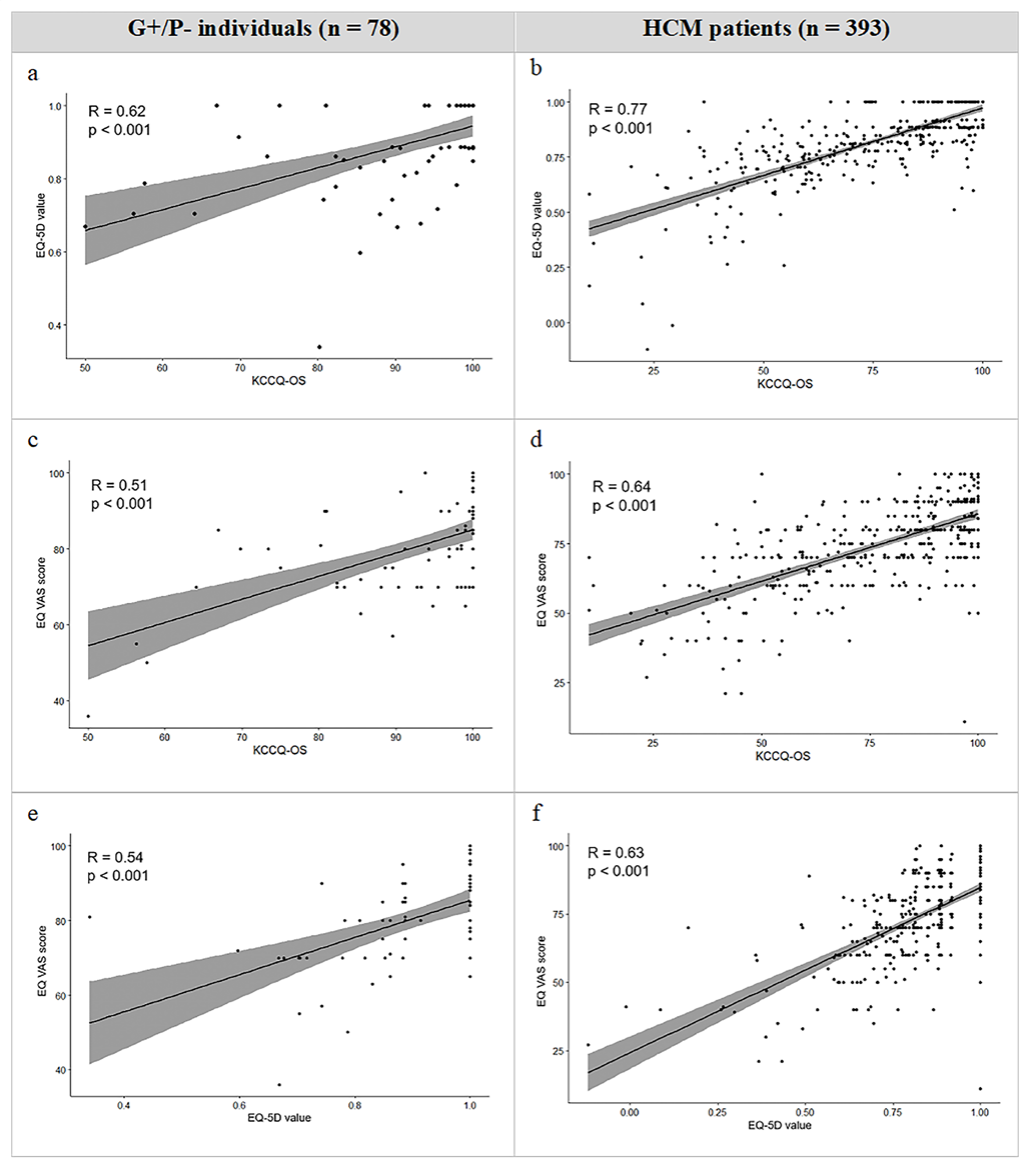
Correlation between EQ-5D values, EQ VAS scores and the KCCQ-OS in G+/P− individuals and HCM patients. Correlation assessed by means of Spearman correlation coefficient. EQ-5D, EuroQoL-5 Dimension; G+/P−, genotype positive, phenotype negative; HCM, hypertrophic cardiomyopathy; KCCQ-OS, Kansas City Cardiomyopathy Questionnaire–Overall Summary Score; VAS, Visual Analogue Scale.

Correlation was highest between the KCCQ-OS and EQ-5D values with a correlation coefficient of 0.62 in G+/P− individuals and 0.77 in HCM patients. Generally, the correlation between instruments was higher in HCM patients and lower in G+/P− individuals. The corresponding correlation scatterplots are visualised in figure 1a–f.

#### Levels of agreement

**Figure 2:**
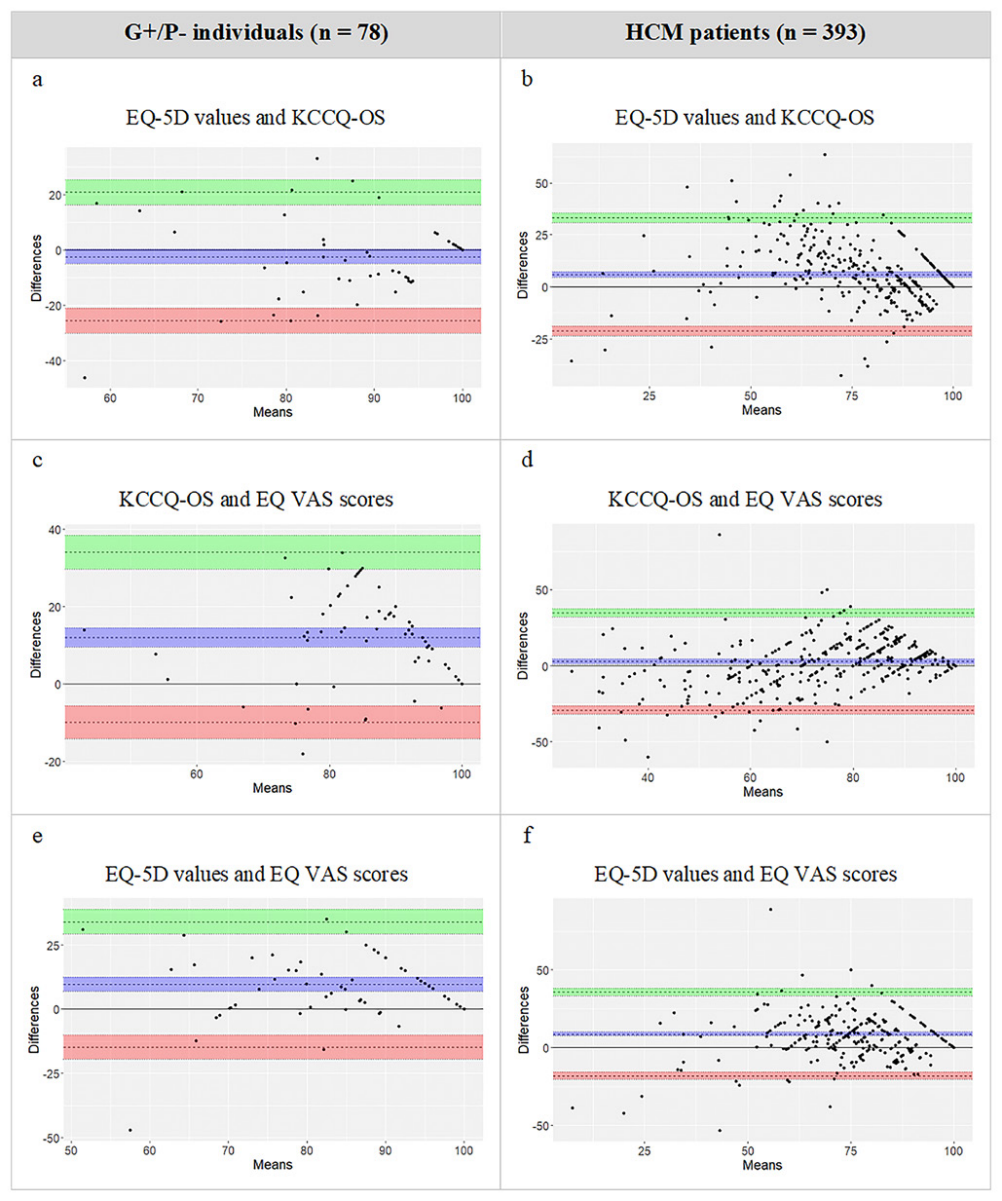
Bland-Altman plots visualising the level of agreement between EQ-5D values, EQ VAS scores and KCCQ-OS in G+/P− individuals and HCM patients. *Bland-Altman plots with zero line (blue) indicating mean difference between instruments and limits of agreement at 1.96 SD away from mean difference. EQ-5D, EuroQoL-5 Dimension; G+/P−, genotype positive, phenotype negative; HCM, hypertrophic cardiomyopathy; KCCQ-OS, Kansas City Cardiomyopathy Questionnaire–Overall Summary Score; VAS, Visual Analogue Scale.

The mean difference between EQ-5D values and the KCCQ-OS was −2.88 in G+/P− and +7.71 in HCM, indicating EQ-5D values being slightly lower in G+/P− individuals and higher in HCM patients compared with KCCQ-OS scores (figure 2a–f). The mean difference between EQ-5D values and KCCQ-OS scores increased to +8.17 in oHCM and +5.23 in nHCM (online supplemental figure S1). Outliers were present in all comparisons. In G+/P− individuals, EQ-5D values and KCCQ-OS scores showed a good level of agreement with an equally distributed pattern and the least number of outliers. In HCM, the pattern was slightly biased with more outliers in the upper limit of agreement. The EQ VAS scored lower than EQ-5D values and KCCQ-OS scores in all subgroups, with highest bias found in G+/P− individuals.

### Construct validity

EQ-5D values, the EQ VAS scores and the KCCQ-OS discriminated between most known groups (table 5). Between G+/P− individuals and HCM patients, all instruments identified significant differences. In asymptomatic versus symptomatic HCM patients and between NYHA I-II and NYHA I-III, differences were found with p<0.001. In nHCM and oHCM, no instrument found a significant difference. When comparing NYHA II-III, the KCCQ-OS found a statistically significant difference (p=0.049), while the EQ-5D values and the EQ VAS scores were slightly above the alpha level of 0.05.

**Table 5:** Construct validity of the EQ-5D values, EQ VAS scores and the KCCQ-OS

Variables used to differentiate groups	Groups (n)	Mean score		
		EQ-5D value	EQ VAS score	KCCQ-OS
G+/P− individuals vs HCM patients	G+/P− (78)	0.902	80.6	92.6
	All HCM (393)	0.836	74.9	77.7
	P value	<0.001	0.003	<0.001
Asymptomatic vsSymptomatic	Asymptomatic (158)	0.901	80.3	88.7
	Symptomatic (235)	0.785	70.5	69.0
	P value	<0.001	<0.001	<0.001
Obstructive HCMNon-obstructive HCM*	Non-obstructive HCM (292)	0.839	75.5	78.7
	Obstructive HCM (101)	0.828	73.2	74.8
	P value	0.3432	0.1526	0.0631
NYHA I vs NYHA II	NYHA I (235)	0.892	79.1	87.5
	NYHA II (134)	0.769	69.7	65.3
	Adjusted p value	<0.001	<0.001	<0.001
NYHA I vs NYHA III	NYHA I (235)	0.892	79.1	87.5
	NYHA III (24)	0.662	62.5	50.4
	Adjusted p value	<0.001	<0.001	<0.001
NYHA II vs NYHA III	NYHA II (134)	0.769	69.7	65.3
	NYHA III (24)	0.662	62.5	50.4
	Adjusted p value	0.050	0.057	0.049

* Test for obstructive HCM versus non-obstructive HCM was two-sided, as no clear direction can be assumed.

EQ-5D value, EuroQoL-5 Dimension; G+/P−, genotype positive, phenotype negative; HCM, hypertrophic cardiomyopathy; KCCQ-OS, Kansas City Cardiomyopathy Questionnaire–Overall Summary Score; VAS, Visual Analogue Scale.

## Discussion

This study performed a psychometric assessment of the generic EQ-5D-5L descriptive system, the EQ VAS and the disease-specific KCCQ in a cohort of G+/P− individuals and HCM patients in the AFFECT-HCM study. The EQ-5D-5L and the KCCQ differed vastly regarding their general characteristics but showed a good overall agreement between EQ-5D values and KCCQ-OS scores. The EQ VAS showed the least level of agreement with other instruments. All instruments discriminated between most known groups, with the KCCQ-OS showing the best differentiation in symptomatic patients.

In the EQ-5D-5L, 50% of the G+/P− individuals reported perfect health. In the EQ-5D-5L, ceiling effects are known and have been reported earlier.^9 16 23^ However, Bland-Altman plots showed that EQ-5D values were on average lower than the disease-specific KCCQ-OS scores, probably because the EQ-5D-5L assesses overall HRQoL whereas the KCCQ has a major focus on heart failure-specific questions which do not apply for most G+/P− individuals.^5^ The high ceiling effects could also mean that G+/P− individuals may simply have no impairments in HRQoL. Christiaans *et al* measured HRQoL with the generic SF-36 in HCM G+/P− individuals and concluded that HRQoL only differed on a minority of subscales from that of the general population.^24^ The AFFECT-HCM study (2024) also showed that G+/P− individuals have comparable-to-better EQ-5D-5L values as the Dutch general population.^18^ Similarly, the mean EQ VAS score of the general population is 80.6, identical to our cohort, suggesting that HRQoL in G+/P− individuals is likely preserved rather than a limited sensitivity of the instruments.^13^

In HCM, the HRQoL of a patient as captured with the EQ-5D value was on average higher compared with the corresponding KCCQ-OS score of the same individual. Further, in NYHA II and III, the KCCQ-OS was the only instrument identifying a significant difference, indicating a better performance of the KCCQ-OS in more symptomatic groups. However, due to the low sample size in NYHA III, it is unclear whether this comparison is robust. No instrument identified a significant difference between nHCM and oHCM, probably because nHCM and oHCM typically cannot be distinguished based on their symptom profile.

In this study, the agreement between EQ-5D values and KCCQ-OS scores was found to be good, indicating that the EQ-5D-5L can assess HRQoL with similar strength to the KCCQ. With the increasing interest in patient-reported outcome measurements, patients are sometimes overloaded with questionnaires. The EQ-5D-5L can be filled out in less than 2 min, making it a short and free alternative to lengthy disease-specific instruments.^25^ Recently, the shorter 12-item version of the KCCQ showed good psychometric properties in oHCM patients, which could also be considered as a shorter (but paid) opportunity for regular HRQoL assessment in clinical practice.^7^

Although the correlation was high, instruments should not be used interchangeably. Thomas *et al* mapped the 12-item and 23-item versions of the KCCQ to the EQ-5D-3L and EQ-5D-5L values in heart failure to estimate societal-based values (utilities) for use in cost-effectiveness analyses.^26^ However, mapping generic to disease-specific HRQoL can be challenging and might not completely represent utilities.^14^ In HCM, no mapping study is available yet, prompting the use of the EQ-5D-5L if scores are intended to be used in health economic evaluations. For clinical trials or a more precise longitudinal monitoring of the patients symptoms and health status, the longer KCCQ may have advantages by providing more insights into the condition and specific health-related subdomains.^5^

This study has several strengths. A thorough comparison of the KCCQ, EQ-5D-5L and EQ VAS has not been conducted previously in HCM and G+/P− individuals. Bootstrapping confirmed the robustness of our results for all instruments. Further, this study provides insights into the strengths and weaknesses of all three instruments, which can guide future research.

Some limitations need to be mentioned. First, we could not assess all psychometric properties of the COSMIN checklist, such as responsiveness (no longitudinal data available yet) and criterion validity (no gold standard for HRQoL instruments).^19 22^ Second, the low sample size in NYHA III made the group comparison between NYHA II and III patients difficult and might limit the generalisability of this result. However, the NYHA distribution of the AFFECT-HCM likely reflects the actual distribution of patients in an outpatient setting.^27^ Third, two negative EQ-5D values were left out of the analysis as those cannot be reported in the scale of the KCCQ and EQ VAS. Due to this very small number, this limitation is relative and is not expected to have a major impact on the results.

In future, similar studies with a comparison of the shorter 12-item version of the KCCQ could provide additional recommendations for routine HRQoL assessment in HCM. Further, prospective research assessing the EQ-5D-5L and other instruments to monitor therapeutic effects in real life outpatient settings and the potential of these instruments to measure these effects in the same patients over time will be necessary. In addition, studies confirming the HRQoL results for G+/P− individuals using the same questionnaires and studies further assessing whether other HRQoL instruments might be more suitable in this specific group could be valuable.

## Conclusions

In HCM, the EQ-5D-5L and the KCCQ had a good agreement and performed well regarding construct validity, with the KCCQ revealing more nuances in symptomatic patients. Given the benefits of the EQ-5D-5L in terms of practicability and its high correlation with the KCCQ, the EQ-5D-5L is a suitable substitute for outpatient use, whereas the KCCQ might be more prudent for clinical research or deep HRQoL assessments. As the EQ-5D-5L and the KCCQ serve different purposes, the final selection of the questionnaires should depend on the individual study objective.

## Supplementary material

10.1136/openhrt-2024-003143 online supplemental file 1

## Data Availability

Data are available on reasonable request.
