# Supplementary material for: Assessing the psychometric properties of generic (EQ-5D-5L) and disease-specific (KCCQ) quality of life in patients with hypertrophic cardiomyopathy in the AFFECT-HCM study
Source: Open Heart. 2025 May 27;12(1):e003143. doi: 10.1136/openhrt-2024-003143 (PMC12121577; doi:10.1136/openhrt-2024-003143)
Supplement: online supplemental file 1 [file openhrt-12-1-s001.docx]

**Supplementary material - Psychometrics of the EQ-5D-5L and KCCQ in HCM**

Table S1 – Baseline characteristics AFFECT-HCM

| **Baseline characteristics** | **G+/P-**  **(n=84)** | **HCM**  **(n=422)** | **P value** |
| --- | --- | --- | --- |
| **General characteristics** |  |  |  |
| Age in years (mean ± SD) | 46 (± 14.8) | 57 (± 13.6) | <.001 |
| Female (n, %) | 57 (68) | 140 (33) | <.001 |
| BMI (kg/m2, mean ± SD) | 25.0 (± 4.6) | 27.5 (± 4.2) | <.001 |
| **Medical history** |  |  |  |
| Hypertension (n, %) | 8 (10) | 148 (35) | <.001 |
| Diabetes mellitus (n, %) | 4 (5) | 43 (10) | 0.118 |
| Dyslipidaemia (n, %) | 3 (4) | 70 (17) | 0.002 |
| CAD (n, %) | 0 | 27 (6) | 0.017 |
| **HCM characteristics** |  | | |
| Age of diagnosis in years  (mean ± SD) | - | 48 (± 15.5) | - |
| P/LP gene variant (n, %) | 84 (100) | 228 (54) | <.001 |
| NYHA classification (n, %) | - |  | - |
| NYHA I |  | 254 (60) |  |
| NYHA II |  | 141 (33) |  |
| NYHA III |  | 27 (6) |  |
| **History of SRT** |  |  |  |
| SRT (n, %) | - | 102 (24) | - |
| Myectomy |  | 84 (82) |  |
| Alcohol septal ablation |  | 15 (15) |  |
| Both |  | 3 (3) |  |
| **History of ICD** |  |  |  |
| ICD (n, %) | 0 | 115 (27) | <.001 |
| Primary prevention ICD |  | 96 (83) |  |

Abbreviations: BMI: body mass index; CAD: coronary artery disease; G+/P-: genotype-positive, phenotype-negative; HCM: hypertrophic cardiomyopathy; ICD: implantable cardioverter defibrillator; nHCM: non-obstructive HCM; NYHA: New York Health Association class; oHCM: obstructive HCM; P/LP: pathogenic/likely pathogenic gene variant; SD: standard deviation; SRT: septal reduction therapy.

Table S2 – Bootstrapped mean scores and 95% confidence interval (CI) of EQ-5D values, EQ VAS scores and KCCQ-OS scores

| **Health-related quality of life - Bootstrapped mean scores** | **n** | **EQ-5D value [95%CI]** | **EQ VAS score  [95%CI]** | **KCCQ-OS  [95%CI]** |
| --- | --- | --- | --- | --- |
| G+/P- individuals | 78 | 0.903 [0.873; 0.929] | 80.6 [77.9; 83.2] | 92.6 [90.0; 95.0] |
| All HCM patients | 393 | 0.836 [0.818; 0.853] | 74.8 [73.2; 76.4] | 77.7 [75.5; 79.9] |
| **HCM subtype** | | | | |
| Non-obstructive HCM | 292 | 0.839 [0.819; 0.858] | 75.5 [73.7; 77.5] | 78.8 [76.3; 81.2] |
| Obstructive HCM | 101 | 0.828 [0.793; 0.859] | 72.9 [69.9; 76.0] | 74.8 [70.5; 78.6] |
| **Symptomatology** | | | | |
| HCM and NYHA I | 235 | 0.892 [0.876; 0.908] | 78.9 [77.2; 80.6] | 87.5 [85.7; 89.4] |
| HCM and NYHA II | 134 | 0.768 [0.735; 0.799] | 69.7 [66.6; 72.5] | 65.1 [61.6; 68.6] |
| HCM and NYHA III | 24 | 0.661 [0.551; 0.749] | 62.4 [55.7; 68.8] | 50.9 [41.8; 59.2] |

Abbreviations: EQ-5D value = value derived from the health profiles of the EQ-5D-5L; EQ VAS score = score reported by individual on the visual analogue scale of the EQ-5D-5L; KCCQ-OS = Kansas City Cardiomyopathy Questionnaire – Overall Summary Score; G+/P- = genotype positive, phenotype negative; HCM = hypertrophic cardiomyopathy; NYHA = New York Heart Association class; CI = confidence interval.

Figures S1a-1f – Bland-Altman plots visualising the level of agreement between EQ-5D values, EQ VAS scores and the KCCQ-OS in non-obstructive and obstructive hypertrophic cardiomyopathy patients

| **nHCM patients (n = 292)** | **oHCM patients (n = 101)** |
| --- | --- |
| Figure 1a  EQ-5D values and KCCQ-OS  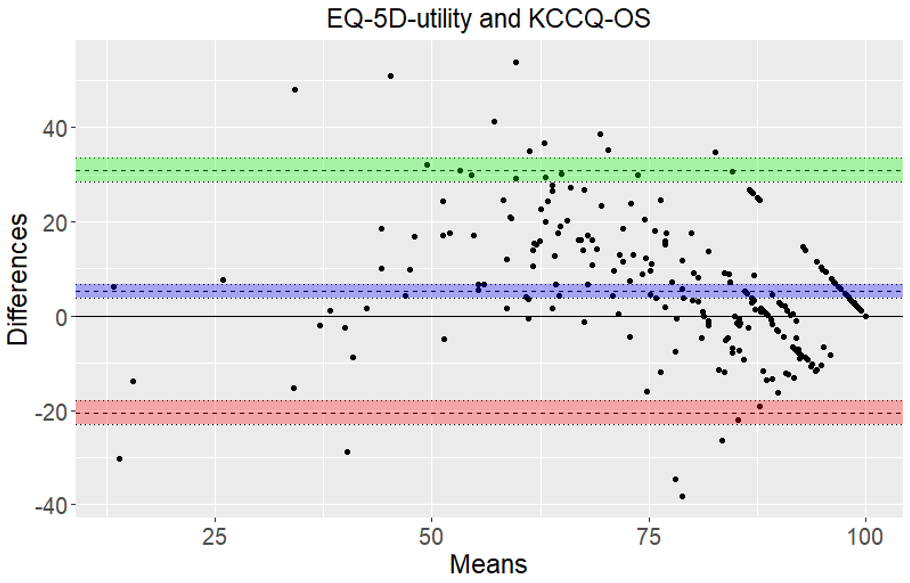 | Figure 1b  EQ-5D values and KCCQ-OS  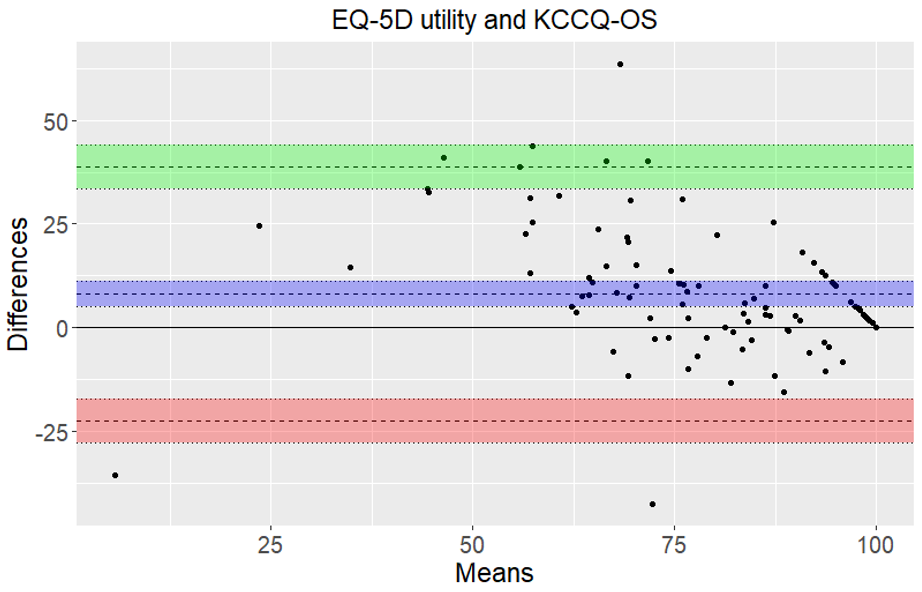 |
| Figure 1c  KCCQ-OS and EQ VAS scores  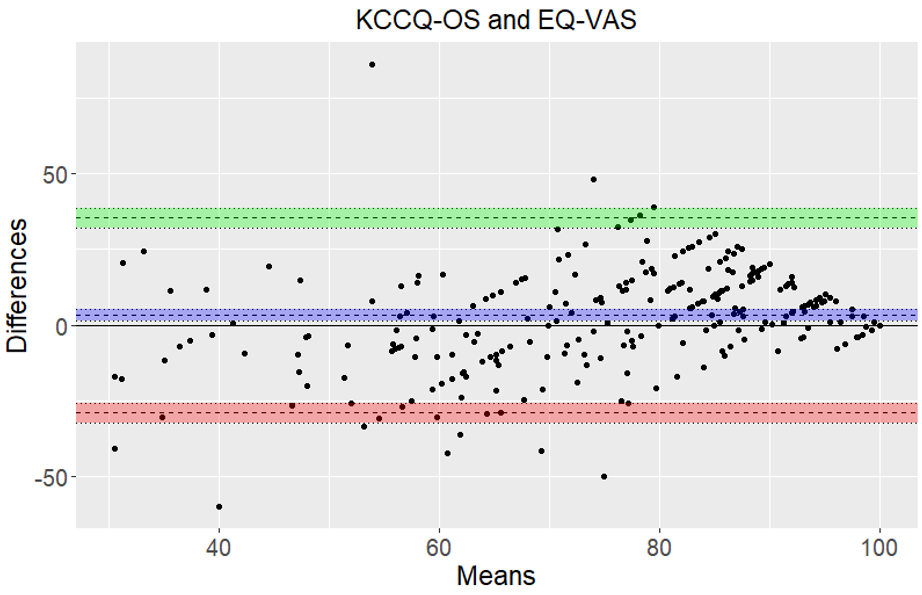 | Figure 1d  KCCQ-OS and EQ VAS scores  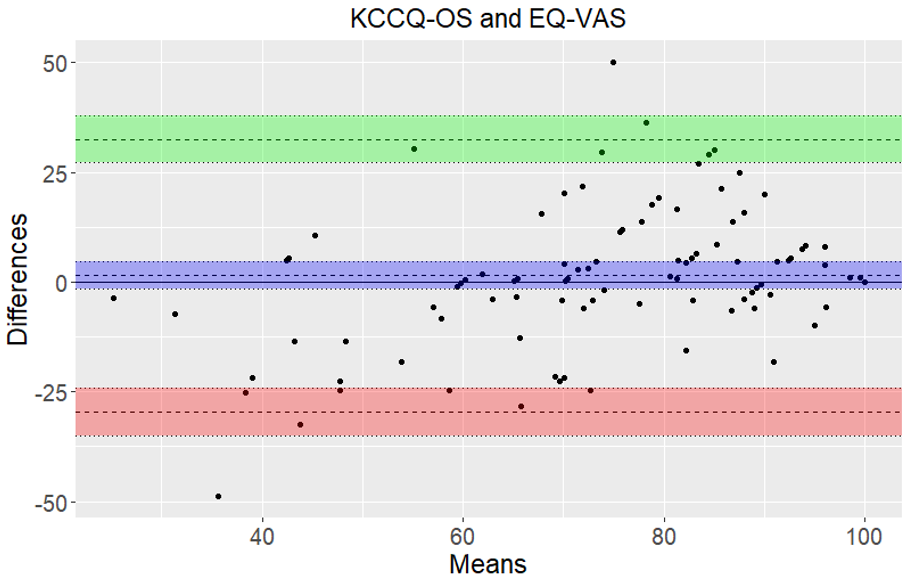 |
| Figure 1e  EQ-5D values and EQ VAS scores  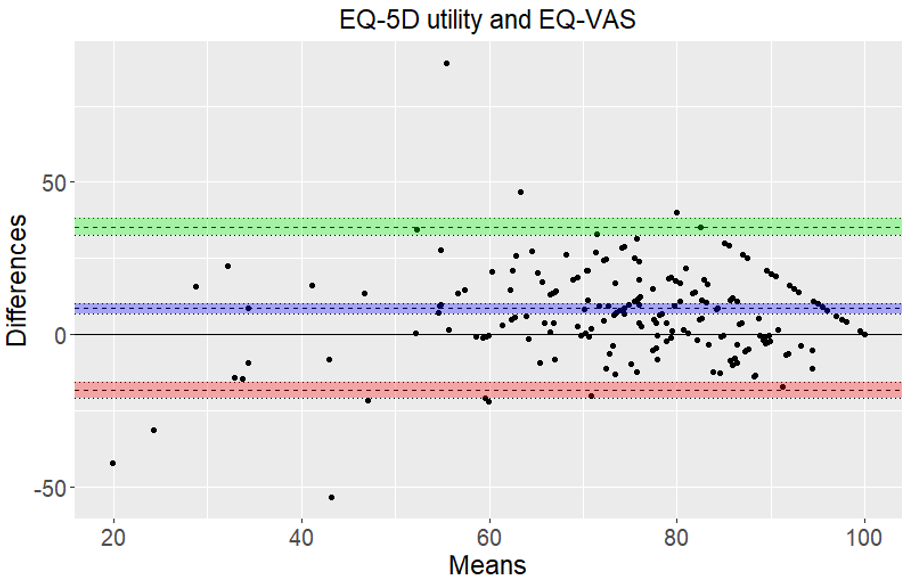 | Figure 1f  EQ-5D values and EQ VAS scores  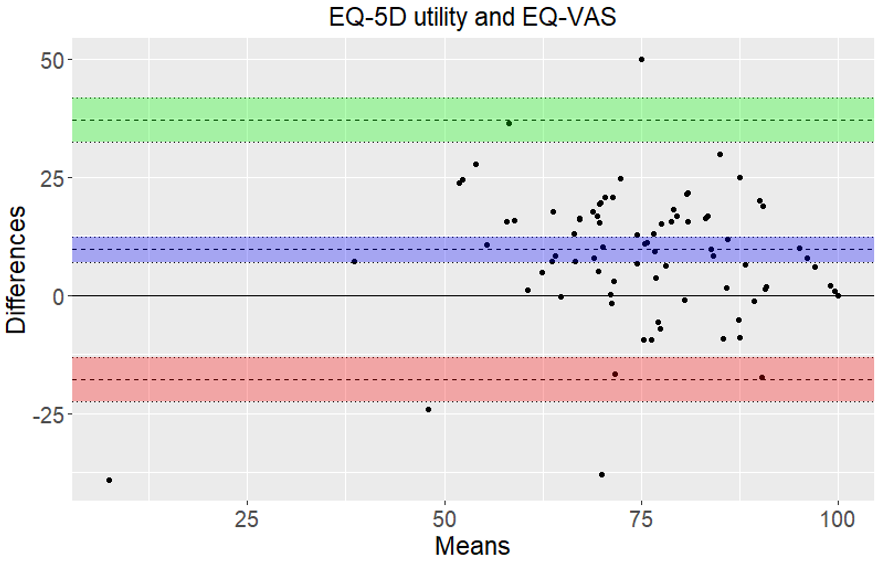 |

Abbreviations: EQ-5D value = values derived from the health profiles of the EQ-5D-5L; EQ VAS score = score reported by individual on the visual analogue scale of the EQ-5D-5L; KCCQ-OS = Kansas City Cardiomyopathy Questionnaire – Overall Summary Score; nHCM = non-obstructive hypertrophic cardiomyopathy; oHCM = obstructive hypertrophic cardiomyopathy.

* Bland-Altman plots with zero line (blue) indicating mean difference between instruments and limits of agreement at 1.96 standard deviations away from mean difference.
